# Ignored by the boss: a moderated-mediation study of boss phubbing

**DOI:** 10.3389/fpsyg.2025.1692595

**Published:** 2026-01-05

**Authors:** Jale Balkaş, Mehmet Taş, Ayşe Günsel, Serdar Bozkurt, Gönül Konakay, Bumin Çağatay Aksu

**Affiliations:** 1 Business Administration Department, Kocaeli University, Kocaeli, Türkiye; 2 Business Administration Department, Yildiz Technical University, Istanbul, Türkiye; 3 Istanbul Gelişim University, Healthcare Management, Istanbul, Türkiye

**Keywords:** boss phubbing, job satisfaction, satisfaction with supervision (JDI), power distance, leadership behavior, workplace relationships

## Abstract

**Introduction:**

This study investigates the workplace implications of boss phubbing (BP)—a managerial behavior in which supervisors prioritize smartphone use over face-to-face interactions with employees. Drawing on Expectancy Violation Theory (EVT), Social Exchange Theory (SET), and Leader–Member Exchange (LMX), BP is conceptualized as a negative relational signal that can undermine employees’ job attitudes.

**Methods:**

A moderated mediation model was tested to examine whether satisfaction with supervision (JDI) mediates the relationship between BP and job satisfaction, and whether power distance moderates the direct and indirect effects. Survey data were collected from 412 full-time employees and analyzed using PROCESS Model 7 with bootstrapping.

**Results and discussion:**

BP significantly reduced satisfaction with supervision (JDI), which in turn fully mediated its effect on job satisfaction; the direct effect of BP on job satisfaction was not significant. Power distance did not moderate the direct path from BP to satisfaction with supervision (JDI) but did moderate the indirect effect, which was stronger for employees low in power distance. Together, these findings indicate that boss phubbing operates primarily through relational exchange processes and that cultural value orientations shape the extent to which inattentive leadership undermines employees’ job attitudes. These results contribute to the growing literature on digital workplace behaviors by clarifying how, and under what conditions, boss phubbing erodes employee outcomes.

## 1 Introduction

Smartphones have become an indispensable part of daily life, enabling seamless access to information and facilitating global communication (Abeele et al., 2019). Their widespread adoption has transformed both social and professional dynamics by fostering frequent interactions and effortless information sharing (Ríos Ariza et al., 2021). While regional variations exist, global smartphone users reached 6.9 billion in 2023, with Turkey reporting an adoption rate of 81.68% (MOBİSAD, 2023). Yet, as digital connectivity increases, concerns about its unintended consequences—particularly the erosion of face-to-face interactions—have intensified (Aagaard, 2020; Abeele et al., 2019).

One manifestation of this phenomenon is phubbing, a fusion of “phone” and “snubbing,” which refers to prioritizing smartphones over direct interpersonal communication (Sun and Samp, 2022). Phubbing disrupts emotional exchange and social engagement, prompting individuals to neglect those physically present, including family, friends, and colleagues (Xie and Xie, 2020; Yurtseven and Duman, 2021). Turkle (2011) highlights how growing reliance on digital devices diminishes face-to-face connections, evoking perceptions of disrespect, contempt, and dissatisfaction (Ríos Ariza et al., 2021).

Phubbing has been linked to internet addiction (Chotpitayasunondh and Douglas, 2016; Karadağ et al., 2015) and a greater propensity among socially excluded individuals to disengage from physical interactions (Roberts and David, 2017). Although its root causes remain under investigation, its detrimental effects on individual well-being and interpersonal relationships are well documented (Erzen et al., 2019; Roberts and David, 2017; Wang et al., 2017). While prior research has extensively examined its psychological and social consequences (Sun and Samp, 2022; Xie and Xie, 2020; Al-Saggaf and MacCulloch, 2019; Karadağ et al., 2015; Wang et al., 2017; Liu et al., 2021), its workplace implications remain comparatively underexplored (Roberts and David, 2017, 2020; Yasin et al., 2023; Khan et al., 2022).

Given that approximately 82% of employees use smartphones during work hours (Career Builder, 2016), understanding workplace phubbing has become increasingly important. Studies highlight the cognitive strain caused by excessive smartphone use in professional settings (Chotpitayasunondh and Douglas, 2016) and its adverse impact on employee well-being and performance (Derks and Bakker, 2014). In this context, researchers have conceptualized boss phubbing (BP) as employees’ perception that their supervisors are distracted by smartphones during interactions (Roberts and David, 2017). This behavior undermines communication, trust, and engagement, creating a disengaging work environment.

From the lens of Expectancy Violation Theory (EVT) (Burgoon, 1993; Burgoon and Le Poire, 1993), BP constitutes a violation of workplace interaction norms, as employees expect their supervisors to provide undivided attention. When these expectations are breached, employees often interpret the behavior as disrespectful and disengaging (Høgh et al., 2021). Such violations can erode supervisor–employee rapport and diminish job satisfaction. Empirical evidence supports this notion, showing that BP negatively affects job satisfaction, employee engagement, and trust in leadership (Roberts and David, 2017, 2020), while also fostering feelings of social exclusion and lower organizational self-esteem among employees (Yasin et al., 2023).

Despite growing interest in BP’s consequences (Roberts and David, 2017; Yasin et al., 2023; Khan et al., 2022), gaps remain in understanding its underlying mechanisms and boundary conditions. This study addresses these gaps by examining:

How employees’ perceptions of BP influence satisfaction with supervision (JDI), ultimately shaping job satisfaction.

How power distance—the extent to which power is unequally distributed (Saputra et al., 2018)—moderates employees’ responses to BP.

By addressing these research questions, this study contributes to the emerging literature on boss phubbing in several ways. First, it extends EVT to the domain of digital workplace behaviors by conceptualizing boss phubbing as a negative expectancy violation that shapes employees’ job attitudes. Second, by integrating EVT with Social Exchange Theory and LMX, the study highlights satisfaction with supervision (JDI) as a key relational mechanism through which boss phubbing affects job satisfaction, thereby foregrounding the role of supervisor–subordinate relationship quality in understanding the consequences of digital distractions at work. Third, by examining power distance as a cultural boundary condition, the study sheds light on when and for whom boss phubbing is most damaging, offering a more nuanced account of how cultural values shape employees’ reactions to inattentive leadership in contemporary organizations.

## 2 Theoretical background and hypothesis development

### 2.1 Boss phubbing: a workplace disruptor

Phubbing, defined as the act of ignoring someone in favor of a smartphone, disrupts interpersonal interactions and erodes relationship quality (Ugur and Koc, 2015). Initially examined in social contexts such as partner phubbing (Roberts and David, 2016), parental phubbing (Xie and Xie, 2020), and friend-related phubbing (Sun and Samp, 2022), the phenomenon also extends into professional environments (Yasin et al., 2023). Across contexts, phubbing consistently undermines communication, reduces engagement, and fosters dissatisfaction (Al-Saggaf and MacCulloch, 2019).

The rise of smartphone dependence has driven extensive research on the antecedents and consequences of phubbing. Studies identify internet addiction, fear of missing out (FOMO), and diminished self-control as key predictors (Chotpitayasunondh and Douglas, 2018). A constant state of being permanently online and connected (POPC) also amplifies phubbing tendencies (Schneider and Hitzfeld, 2021). Beyond interpersonal costs, phubbing is associated with depression (Wang et al., 2017), lower life satisfaction (Çikrikci et al., 2019), reduced relationship quality (Sun and Samp, 2022), and psychological distress (Shahbaz et al., 2020).

In organizational settings, boss phubbing (BP)—when supervisors prioritize smartphone use over employee interactions—has emerged as a significant workplace disruptor (Roberts and David, 2017, 2020; Khan et al., 2022; Hasan et al., 2024). Given that employees spend a substantial portion of their time at work, the implications of BP are profound, affecting job satisfaction, motivation, and performance (Yasin et al., 2023). As smartphones become more embedded in professional routines, their ubiquity introduces both efficiency gains and unintended relational costs (Roberts and David, 2020).

BP is increasingly viewed as a counterproductive workplace behavior, weakening employee–supervisor relationships by fostering disengagement and dissatisfaction (Roberts and David, 2017). It is not merely a social nuisance; it actively undermines workplace morale and productivity (Yuda and Suyono, 2024). Studies consistently demonstrate that BP diminishes work engagement and job performance, with intrinsic motivation often serving as a mediating mechanism (Yousaf et al., 2022). Employees exposed to BP report lower perceived supervisor support, weaker professional identification, and reduced performance outcomes (Bracht et al., 2024).

Earlier studies established BP’s negative impact on workplace trust and employee engagement (Roberts and David, 2017), while subsequent research confirmed its detrimental effects on job satisfaction and organizational commitment (Roberts and David, 2020). Recent evidence further links BP to workplace incivility, presenteeism, and lower subordinate identification with supervisors (Khan et al., 2023; Hasan et al., 2024). BP has also been associated with declines in employees’ psychological capital and work–family enrichment (Zhen and Wen, 2022; Yao and Nie, 2023).

Overall, the growing literature underscores BP’s far-reaching consequences for employee attitudes, workplace cohesion, and organizational effectiveness (Yuda and Suyono, 2024). Yet, despite these insights, gaps remain in understanding the mechanisms and boundary conditions underlying these effects. Addressing these gaps is essential to developing evidence-based interventions that can mitigate BP’s disruptive influence and promote healthier workplace dynamics.

### 2.2 Boss phubbing and satisfaction with supervision (JDI)

Expectancy Violation Theory (EVT) posits that individuals develop implicit behavioral expectations in social interactions, particularly concerning nonverbal engagement (Burgoon et al., 1989). When these expectations are violated—such as when a supervisor diverts attention to a smartphone—the interaction is disrupted, prompting the observer to reassess both the behavior and its underlying intent (Burgoon and Hale, 1988). This evaluative process involves two steps: first, interpreting the behavior’s significance, and second, assessing its desirability in the given context. While positive expectancy violations may enhance communication and trust, negative violations—such as inattentiveness—undermine rapport and engagement (Burgoon et al., 1989).

In professional settings, employees expect supervisors to provide full engagement during communication (Burgoon et al., 1989). When supervisors divert attention to smartphones, employees often perceive the behavior as signaling a lack of value or respect toward them and their contributions (Roberts and David, 2020; Anshari et al., 2016; Yousaf et al., 2022). Such violations weaken relational trust and diminish communication effectiveness, as supported by research on nonverbal intimacy in workplace interactions (Brandt, 1979). Conversely, when supervisors display attentiveness and warmth, employees report higher satisfaction and stronger professional relationships (Castañeda and Nahavandi, 1991). In contrast, the absence of nonverbal intimacy increases the likelihood of negative supervisor evaluations and reduced satisfaction (Teven, 2007).

Satisfaction with supervision (JDI) refers to employees’ affective evaluation of their supervisor’s interpersonal behavior and treatment (perceived respect, fairness, sup-portiveness, and communication quality) during work interactions (Teven, 2007; García-Cabrera et al., 2023; Singh, 2024; Roy et al., 2025). Empirical studies demonstrate that phubbing undermines interpersonal communication, weakens relational bonds, and fosters dissatisfaction (Erzen et al., 2019; Roberts and David, 2016, 2017; Wang et al., 2017; Ríos Ariza et al., 2021; Yurtseven and Duman, 2021). In supervisory contexts, phubbing erodes perceived communication quality, lowering satisfaction with supervision (JDI) (Chotpitayasunondh and Douglas, 2018). These findings align with broader evidence emphasizing that nonverbal intimacy is crucial for cultivating positive supervisor–subordinate relationships, and its absence diminishes perceived leadership effectiveness (Teven, 2007). Thus, we hypothesize:

*H1*: Boss phubbing is negatively associated with satisfaction with supervision (JDI).

### 2.3 Boss phubbing and job satisfaction

Job satisfaction is defined as an emotional response to the alignment—or misalignment—between employees’ expectations and their actual work experiences (Locke, 1969). When workplace conditions meet or exceed expectations, employees report higher satisfaction; when expectations are unmet, dissatisfaction and disengagement follow. Organizational psychology literature highlights that job satisfaction is shaped by both specific job facets (e.g., supervisor interactions, compensation) and overall work experiences (Brayfield and Rothe, 1951; Smith et al., 1969). Some scholars conceptualize job satisfaction as a multidimensional construct influenced by factors such as supervisor relationships, organizational culture, leadership style, and the social work environment (Schwepker, 2001; Han and Jekel, 2011; Tepret and Tuna, 2015). Among these, supervisor–subordinate communication is a critical determinant. Nonverbal cues such as eye contact, facial expressions, and body language play a central role in shaping employees’ perceptions of leadership engagement (Mishra, 2013). Supervisors influence employees’ workplace experiences by providing guidance, articulating organizational objectives, and delivering performance feedback (Steele and Plenty, 2014). However, when supervisors appear distracted—frequently engaging with their smartphones during interactions—their perceived attentiveness declines, eroding trust and job satisfaction.

The link between supervisor communication and job satisfaction is well-established. High-quality leader–employee interactions foster job satisfaction (Miles et al., 1996; Babalola, 2016; Stringer, 2006), while dismissive or condescending communication elicits negative responses (Czech and Forward, 2013). Similarly, trust in leadership (Dirks and Ferrin, 2001), emotional support from supervisors (Pohl and Galletta, 2017), and two-way communication (Van der Wal et al., 2016) are all key drivers of job satisfaction.

Boss phubbing, by prioritizing smartphone use over employee interaction, undermines trust, damages relational quality, and reduces job satisfaction (Roberts and David, 2020). Employees subjected to BP frequently feel undervalued, disengaged, and less committed to their roles, ultimately harming both morale and organizational effectiveness. Thus, we hypothesize:

*H2*: Boss phubbing is negatively associated with job satisfaction.

### 2.4 Satisfaction with supervision (JDI) and job satisfaction

Supervisors and managers play a central role in shaping employees’ job satisfaction, a topic extensively examined in organizational behavior (OB) research. Prior studies have highlighted the impact of supervisory behavior (Adebayo and Ogunsina, 2011), leadership style (Rowold et al., 2014), supervisor support (Alegre et al., 2016), and supervisor–employee relationship quality (Stringer, 2006). Among these dimensions, satisfaction with supervision (JDI) consistently emerges as a primary determinant of overall job satisfaction (Platis et al., 2015). Supervisors influence the work environment by facilitating communication, providing performance feedback, and modeling workplace engagement, all of which significantly shape employee attitudes (Griffin et al., 2001). When supervisors demonstrate attentiveness and maintain open, supportive communication, employees report higher levels of job satisfaction.

Extensive research supports a strong association between satisfaction with supervision (JDI) and positive job-related attitudes, including organizational commitment (DeConinck and Stilwell, 2004). Supervisory behaviors characterized by friendliness, empathy, and approachability foster employee satisfaction (Locke, 1969; Griffin et al., 2001). Employees interpret the quality of supervisory communication and engagement as a reflection of managerial support, which directly influences job satisfaction and commitment (Pohl and Galletta, 2017). Conversely, negative supervisory behaviors—such as disengagement, inattentiveness, or lack of support—undermine satisfaction and reduce motivation. Leadership behaviors, managerial discretion, and communication style collectively determine the strength of the supervisor–subordinate relationship and its effect on job satisfaction (van der Wal et al., 2016). Based on this evidence, supervisors who exhibit supportiveness, accessibility, and authenticity are expected to cultivate higher employee satisfaction. Thus, we hypothesize:

*H3*: Satisfaction with supervision (JDI) is positively associated with job satisfaction.

### 2.5 Mediator role of satisfaction with supervision (JDI) in boss phubbing-job satisfaction relationship

Job satisfaction is widely recognized as a multifaceted evaluation shaped not only by structural features of the job but also by the quality of relationships in the workplace (Belias and Koustelıos, 2014; Çakmak et al., 2015; Mihalcea, 2014; Riaz and Haider, 2010). In particular, the way supervisors interact with employees—whether they are perceived as attentive, respectful, and supportive—plays a central role in shaping employees’ overall job attitudes.

Social Exchange Theory (SET) conceptualizes the employment relationship as an ongoing series of resource exchanges in which parties reciprocate favorable or unfavorable treatment over time (Blau, 1964; Cropanzano and Mitchell, 2005). When employees experience attention, respect, and support from their supervisors, they are more likely to reciprocate with positive attitudes such as satisfaction and commitment. Conversely, signals of neglect or disrespect reduce employees’ willingness to reciprocate and can erode their evaluations of both the relationship and the job.

Leader–Member Exchange (LMX) theory applies this relational perspective to supervisor–subordinate dyads, emphasizing that leaders develop differentiated relationships with followers that vary in mutual trust, respect, and obligation (Graen and Uhl-Bien, 1995). High-quality LMX, characterized by frequent, open communication and perceived socioemotional support, is robustly associated with higher job satisfaction and stronger organizational commitment, whereas low-quality exchanges are linked to dissatisfaction and withdrawal (Han and Jekel, 2011). From this standpoint, employees’ satisfaction with supervision (JDI) represents a central affective indicator of the perceived quality of the exchange relationship.

Building on EVT, BP can be understood as a negative relational signal within these exchange processes. When supervisors divert their attention from employees to smartphones during interactions, they not only violate expectations of attentiveness but also convey low relational value—signaling that the employee and the interaction are not worth undivided attention (Roberts and David, 2017, 2020; Yao and Nie, 2023). In SET and LMX terms, such behavior is likely to be interpreted as a lack of relational investment and a degradation in the quality of the leader–member relationship. Over time, repeated exposure to BP should therefore undermine employees’ satisfaction with supervision (JDI). Because satisfaction with supervision (JDI) is a key conduit through which leader behaviors shape global job attitudes (DeConinck and Stilwell, 2004; Stringer, 2006), reductions in satisfaction with supervision (JDI) are expected, in turn, to translate into lower job satisfaction. Thus, we hypothesize:

In organizational settings, employees expect their supervisors to demonstrate attentiveness and interpersonal respect during face-to-face interactions. Boss phubbing (BP)—where a supervisor diverts attention to a mobile device during communication—is perceived as a breach of this normative expectation. According to Expectancy Violation Theory (EVT), deviations from anticipated communicative behavior often lead to negative social evaluations (Bano et al., 2024). When supervisors prioritize their smartphones over employee interactions, this behavior is interpreted as a failure to uphold norms of workplace courtesy. Consequently, employees may feel dismissed or devalued, perceiving such conduct as disrespectful and unjust. The perception of being ignored or taken lightly violates not only communicative expectations but also undermines the employee’s sense of being treated with fairness and dignity.

This perceived disrespect can also thwart the fulfillment of employees’ core socio-relational needs, such as the desire for belonging and connection. When a manager’s inattentiveness signals disregard, employees may experience exclusion, weakening their perceived inclusion within the workplace (Yasin, 2021). The inability to satisfy this need for relatedness fosters emotional detachment, reducing employees’ sense of value and diminishing their satisfaction with their supervisor. Over time, deteriorating satisfaction with supervision (JDI) can erode overall job satisfaction, given that managerial relationships play a central role in shaping employees’ affective evaluations of work. Any perception of interpersonal injustice or lack of recognition within this relationship can negatively influence broader work attitudes. Empirical research supports this view, showing that disruptions in satisfaction with supervision (JDI) adversely affect job satisfaction and, by extension, performance outcomes (Roberts and David, 2017). Thus, BP sets off a chain reaction—beginning with expectation violation and culminating in diminished job satisfaction—by obstructing the psychological mechanisms that support respectful, fair, and socially fulfilling supervisory relationships.

*H4*: Satisfaction with supervision (JDI) mediates the relationship between boss phubbing and job satisfaction.

*H5*: The relationship between boss phubbing and satisfaction with supervision (JDI) is moderated from power distance.

### 2.6 Moderated-mediation: the role of power distance

Power distance also has critical implications for the indirect effect of BP on job satisfaction through satisfaction with supervision (JDI). Cultural norms shape employees’ expectations of leadership behavior, which in turn influence their reactions to supervisory actions (Burgoon et al., 1989). For example, Wang and Fränti (2022) identified five themes through which power distance shapes workplace interactions: hierarchy, authority, communication openness, promotion, and unequal treatment. These findings emphasize how power distance affects the evaluation of managerial behaviors.

BP, characterized by a supervisor’s smartphone distraction during employee interactions, violates expectations of attentiveness and respect (Roberts and David, 2020). According to EVT, such violations trigger negative judgments and emotional responses, weakening supervisor–employee relationships (Burgoon et al., 1989). The severity of these negative reactions, however, depends on cultural context.

In low power distance cultures, employees expect more engaged and egalitarian interactions with supervisors. BP is more likely to be perceived as a salient norm violation, producing stronger negative emotional reactions and reducing satisfaction with supervision (JDI). In contrast, high power distance cultures feature lower expectations of direct engagement; as a result, BP may be interpreted as less severe, and its negative consequences are likely to be mitigated.

Given that satisfaction with supervision (JDI) mediates the relationship between BP and job satisfaction, we propose that power distance moderates this indirect effect. Specifically, the negative mediated pathway is expected to be stronger in low power distance cultures, where employees value participatory leadership, and weaker in high power distance cultures, where hierarchical norms reduce expectations of personal attention. Thus, we hypothesize:

*H6*: Power distance moderates the indirect relationship between boss phubbing and job satisfaction, through satisfaction with supervision (JDI), such that the indirect effect is stronger in low power distance cultures and weaker in high power distance cultures.

**Figure 1:**
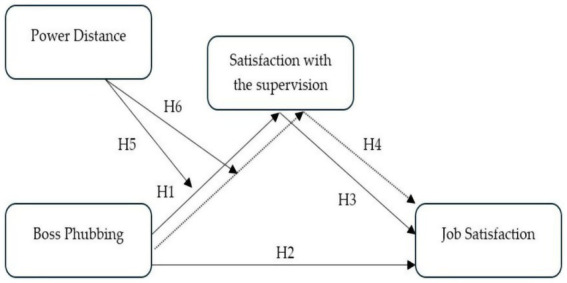
Research model.

The research model corresponding to the aforementioned hypotheses is presented in Figure 1.

## 3 Materials and methods

### 3.1 Measures

To test the study’s hypotheses, multi-item scales derived from established scholarly research were employed to measure the various constructs under investigation. Data were collected through questionnaire surveys using a convenience sampling approach. Respondents rated each item on a 5-point Likert scale, ranging from “Strongly Disagree” (1) to “Strongly Agree” (5), indicating their level of agreement.

BP was measured using the 9-item Boss Phubbing Scale developed by Roberts and David (2017) and adapted to Turkish by Özdemir (2020). Example items from this scale are:“During a typical meeting where my boss and I are both present, my boss pulls out and checks his/her cell phone.,” “My boss places his or her cell phone where I can see it when we are together”.

Boss phubbing (BP) was assessed with an item set capturing attentional division during face-to-face interactions. Items cover both mere visibility/availability (e.g., the phone is placed on the table) and active engagement during interaction (e.g., checking/typing while we talk). Consistent with EVT, our operationalization focuses on the subordinate’s perception of divided attention rather than the supervisor’s intention. To address the content-validity concern that visibility may not imply interference, we re-estimated all models after dropping the visibility-only item (BP2). Results were materially unchanged; measurement quality (loadings, CR/AVE, fit) and the pattern of structural paths remained the same.

Job satisfaction and satisfaction with supervision (JDI) were assessed using the “Job Descriptive Index” developed by Smith et al. (1969), consisting of 9 and 6 items, respectively. Job satisfaction items include: “My work satisfies me.,” “I am not satisfied with my job in general.” satisfaction with supervision (JDI) items include: “My supervisor is rude.,” “My supervisor is annoying.”

Consistent with this definition and to avoid construct–measure mismatch, we operationalized satisfaction with supervision using the JDI Supervision facet; its items index interpersonal treatment (courtesy/respect), fairness, support and feedback, competence, and accessibility rather than pure communication satisfaction (Smith et al., 1969).

To measure power distance, a five-item scale adapted from Brockner et al. (2001) was utilized. Sample items from this scale include: “Communications with superiors should always be done using formally established procedures.,” “People are better off not questioning the decisions of those inauthority.”

### 3.2 Sampling

This study aims to investigate the intricate relationships between boss phubbing, job satisfaction, and satisfaction with supervision (JDI), while also considering the moderating effect of power distance.

To empirically test the proposed hypotheses, a sample of 500 employees from various sectors in Kocaeli/Turkey was selected based on accessibility. Initial contact was made via phone, where the purpose of the study was clearly explained to all potential participants. Of the 500 individuals approached, 458 agreed to take part in the research. Subsequently, 426 participants completed the survey. After a thorough review of the collected data, incomplete responses were excluded, resulting in a final sample of 412 valid responses for further analysis.

Table 1 provides an overview of the demographic characteristics of the study participants. The age distribution reveals that the majority of respondents belong to the younger demographic, with 57.3% aged between 26 and 35 years. This is followed by those aged 36–45 years (18.0%) and 25 years and under (17.5%). Older age groups are less represented, with 6.1% aged 46–55 years and only 1.2% aged 55 years and above. This distribution indicates that the sample is predominantly composed of individuals in early to mid-career stages.

**Table 1:** Demographics variables.

Variables	Category	Frequency	Percent
Age	25 and under	72	17.5%
	26–35	236	57.3%
	36–45	74	18.0%
	46–55	25	6.0%
	55 and more	5	1.2%
Gender	Male	259	62.9%
	Female	152	36.9%
	Unresponse	1	0.2%
Education	Primary School	0	0%
	High School	30	7.3%
	Vocational School	57	13.8%
	Bachelor’s Degree	239	58.0%
	Master’s/Doctorate	86	20.9%
Total tenure	5 years and less	178	43.2%
	6–10 years	106	25.7%
	11–15 years	55	13.4%
	16–20 years	38	9.2%
	21 years and over	35	8.5%
Marital status	Married	204	49.5%
	Single	198	48.1%
	Divorced	10	2.4%
Total		412	100.0

The gender distribution shows an imbalance, with 62.9% of respondents identifying as male and 36.9% as female. This gender disparity may influence the diversity of perspectives captured in the data, highlighting the need to consider gendered implications in the analysis.

Educational attainment among participants is significantly high, with 58.0% holding a bachelor’s degree and 20.9% having completed postgraduate education, including master’s and doctoral degrees. A smaller proportion of respondents have vocational school qualifications (13.8%) or a high school diploma (7.3%), while none reported primary school as their highest level of education. This suggests that the sample is highly educated, with a substantial emphasis on higher education qualifications.

In terms of workforce tenure, the data indicates that 43.2% of respondents have 5 years or less of work experience, signifying a strong presence of early-career professionals. This group is followed by those with 6–10 years of experience (25.7%), 11–15 years (13.3%), 16–20 years (9.2%), and 21 years or more (8.5%). This distribution underscores the dominance of individuals with relatively limited professional experience in the sample.

Marital status is almost evenly distributed, with 49.5% of respondents married and 48.1% single. A smaller proportion (2.4%) reported being divorced. This near parity reflects the diverse life stages of participants included in the study. The survey included a total of 412 respondents, predominantly comprising young, highly educated, and male participants.

### 3.3 Data analysis

A variety of statistical techniques were employed for data analysis, utilizing SPSS and AMOS software. To assess the reliability and validity of the research model, composite reliability (CR), Cronbach’s alpha, and average variance extracted (AVE) were calculated. Additionally, confirmatory factor analysis (CFA) was conducted to evaluate model fit indices, including CMIN/DF, CFI, IFI, TLI, and RMSEA. Moderation and mediation effects were examined using the PROCESS macro (Hayes, 2013). Hypothesis testing and the significance of indirect effects were analyzed through bootstrapping procedures, as outlined by Shrout and Bolger (2002).

## 4 Results

### 4.1 Measurement tests

Confirmatory factor analysis was performed with IBM AMOS 22 to evaluate the proposed measurement model. The findings showed that the model was satisfactory, and the values were within the threshold values (Hair et al., 2010). The values of the model fit indices (see Table 2) indicating a good fit to the data were as follows: CMIN/DF = 2.996 *p* < 0.001; TLI = 0.890; IFI = 0.906; CFI = 0.905, NFI = 865, TLI = 0.849 and RMSEA = 0.070.

**Table 2:** Model fit.

*χ*^2^	df	*χ*^2^/df	RMSEA	CFI	TLI	NFI	RFI
66491.306	164.000	2.996	0.70	0.905	0.890	0.865	0.849

The reliability and validity of the scales were measured by examining Cronbach’s alpha, AVE, CR values, and factor loading (Fornell and Larcker, 1981; Hair et al., 2010). Table 3 shows that Cronbach’s alpha, CR, and AVE values exceeded the threshold values. The convergent validity of each construct was measured through factor loading values. Table 2 shows that the factor loading of each item exceeded 0.5, achieving convergent validity. Also, discriminant validity was assessed in accordance with Fornell and Larcker (1981), who suggested that the values of each inter-correlation measure should be less than the square root of the AVE for each measure. As shown in Table 3, the correlations between all constructs are less than the square root of the AVE, confirming the discriminant validity of the model.

**Table 3:** Measurement of reliability and validity.

Consructs	Items	Loading (unstd.)	SE	CR(t)	*p*	Loading (std.)	CR	AVE	Cronbach’s Alpha
Boss phubbing	BP1					0.635	0.880	0.513	0.878
	BP3	0.906	0.080	0.276	***	0.653			
	BP4	1.019	0.088	0.629	***	0.679			
	BP5	1.263	0.093	0.564	***	0.836			
	BP6	0.948	0.081	0.777	***	0.690			
	BP8	1.091	0.087	0.600	***	0.753			
	BP9	0.998	0.080	0.487	***	0.744			
Satisfaction with the supervision	SS1					0.742	0.872	0.533	0.871
	SS2	1.237	0.118	0.529	***	0.740			
	SS3	0.703	0.074	0.647	***	0.725			
	SS4					0.642			
	SS5	0.810	0.085	0.663	***	0.764			
	SS6	0.990	0.085	0.710	***	0.760			
Job satisfaction	JS1	0.863	0.079	0.929	***	0.698	0.771	0.490	0.767
	JS2					0.762			
	JS3	1.044	0.072	0.430	***	0.572			
	JS6	0.925	0.065	0.131	***	0.666			
Power distance	PD1	0.834	0.067	0.464	***	0.749	0.769	0.536	0.751
	PD2	1.028	0.069	0.905	***	0.879			
	PD3	1.056	0.071	0.833	***	0.524			

SE, Standard error; CR(t), Critical ratio; CR, Composite reliability; AVE, Avarage variance extracted. ***p < 0.001.

Table 4 presents the correlations between the variables, highlighting several significant relationships. Boss phubbing demonstrates a negative correlation with both satisfaction with supervision (JDI) (*r* = −0.459, *p* < 0.01) and job satisfaction (*r* = −0.201, *p* < 0.01), while showing a positive correlation with power distance (*r* = 0.135, *p* < 0.05). Furthermore, satisfaction with supervision (JDI) is positively correlated with job satisfaction (*r* = 0.380, p < 0.01) and negatively correlated with power distance (*r* = −0.162, *p* < 0.01). These correlation results align with the anticipated directions of the relationships among the variables.

**Table 4:** Descriptive statistics, correlation matrix and discriminant validity.

	Mean	SD	1	2	3	4
1. Boss phubbing	3.157	1.330	(0.716)			
2. Satisfaction with the supervision	3.621	1.315	−0.459**	(0.730)		
3. Job satisfaction	4.447	1.092	−0.201**	0.380**	(0.700)	
4. Power distance	5.150	0.874	0.135*	−0.162**	−0.063	(0.732)

*p < 0.05; **p < 0.01.

The Boss Phubbing Scale was intended to assess supervisors’ interaction-disrupting smartphone use during face-to-face exchanges. Although most items capture explicit attention-diverting behaviors, BP2 (My boss places his or her cell phone where I can see it when we are together) represents a comparatively weaker form of the construct. Placing a phone within view does not necessarily imply active attentional division; it may simply reflect a supervisor’s desire to remain reachable for potential notifications. As such, BP2 can be viewed as capturing a boundary condition of phubbing behavior.

Given concerns that BP2 may not fully reflect attentional disruption, we conducted a robustness check by rerunning all analyses with BP2 removed. Reliability coefficients, factor loadings, and the substantive pattern of relationships among variables showed no meaningful changes. Thus, the exclusion of BP2 did not alter the overall interpretation of the findings.

### 4.2 Hypotheses test

The bootstrapping analysis was conducted using the PROCESS macro for SPSS (Hayes, 2012). This method is particularly effective for estimating direct, indirect, and moderated effects within a single analytical framework. It facilitates simultaneous testing of the overall model, incorporating both mediation and moderation, to assess the conditional nature of indirect effects (Edwards and Lambert, 2007; Preacher et al., 2007). Specifically, the technique developed by Preacher et al. (2007) was employed to test the mediating effect of satisfaction with supervision (JDI) on the relationship between boss phubbing and job satisfaction.

Consistent with recent methodological guidance on the use of control variables, we did not include additional demographic or job-related control variables in the PROCESS models. Our theorizing specifies a focal attitudinal process linking boss phubbing to job satisfaction via satisfaction with supervision (JDI) and power distance, and we found no strong theoretical basis to treat variables such as age, gender, tenure, or education as core confounds of these relationships. In line with recent methodological guidance, we therefore avoided *ad hoc* “kitchen-sink” specifications and prioritized a parsimonious, theory-driven model, as the mechanical inclusion of controls can introduce bias, reduce statistical power, and obscure interpretation (Becker et al., 2016; Bernerth and Aguinis, 2016; Spector and Brannick, 2011). We report the results of this focal model and encourage future research to examine context-specific controls where clear theoretical rationales can be articulated.

Table 5 summarizes the regression analyses evaluating the direct, indirect, and mediated relationships among boss phubbing, satisfaction with supervision (JDI), and job satisfaction. The results demonstrate that boss phubbing is significantly and negatively associated with satisfaction with supervision (JDI) (*β* = −0.4051, SE = 0.0445, *t* = −9.0962, *p* < 0.001), thereby supporting H1.

**Table 5:** Regression results for simple mediation.

Variable	*β*	SE	*t*	*p*
Direct and total effects				
Job satisfaction regressed on boss phubbing	0.1320**	0.0400	−3.2993	0.0011
Satisfaction with the supervision regressed on boss phubbing	0.4051**	0.0445	−9.0962	< 0.001
Job satisfaction regressed on satisfaction with the supervision, controlling for boss phubbing	0.2512**	0.0427	5.8904	< 0.001
Job satisfaction regressed on boss phubbing, controlling for satisfaction with the supervision	−0.0320	0.0422	0.7580	0.4738

Value	*M*	SE	LCL 95%CI	UCL 95%CI
Bootstrap results for indirect effect				
Value	−0.1018	0.0226	−0.1476	−0.0587

*N* = 412. Unstandardized regression results are reported. Bootstrap sample size = 5,000. LCL, lower confidence limit; CI, confidence interval; UCL, upper confidence limit. **p < 0.01.

Additionally, a significant negative direct relationship is observed between boss phubbing and job satisfaction (*β* = −0.1320, SE = 0.0400, *t* = −3.2993, *p* = 0.0011). However, when satisfaction with supervision (JDI) is introduced as a mediator, the direct relationship between boss phubbing and job satisfaction weakens and becomes non-significant (*β* = −0.0320, SE = 0.0422, *t* = −0.7170, *p* = 0.4738). This finding indicates the mediating role of satisfaction with supervision (JDI) in this relationship, thus confirming H4, while not supporting H2.

Furthermore, satisfaction with supervision (JDI) exhibits a positive and significant association with job satisfaction, even when controlling for boss phubbing (*β* = 0.2512, SE = 0.0427, *t* = 5.8904, *p* < 0.001), providing strong evidence for H3.

The significance of the indirect effect of boss phubbing on job satisfaction, mediated by satisfaction with supervision (JDI), is further validated through bootstrap analysis. The indirect effect [*β* indirect = −0.1018, SE = 0.0226, 95% CI (−0.1476, −0.0587)] does not include zero, confirming the robustness of the mediation effect. Collectively, these findings underscore the critical role of satisfaction with supervision (JDI) as a mediating variable, fully mediating the relationship between boss phubbing and job satisfaction.

**Figure 2:**
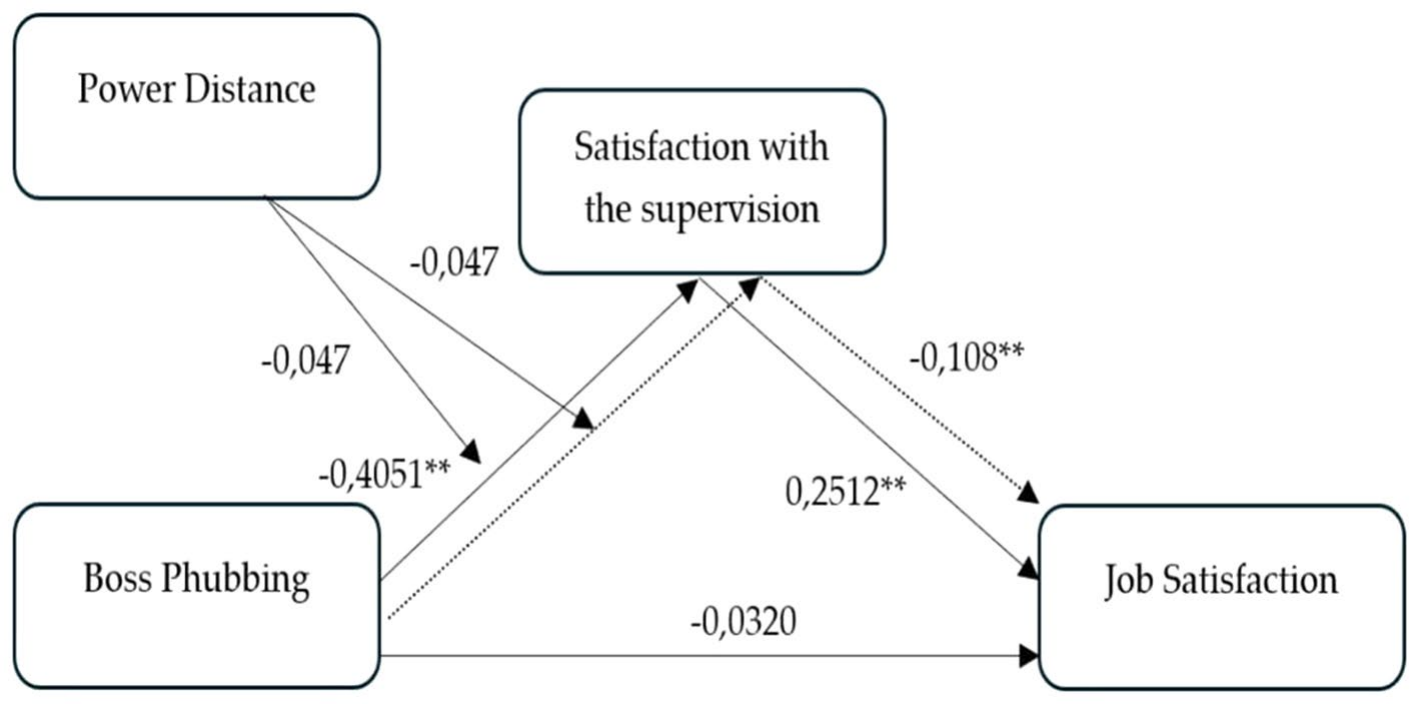
Results.

Table 6 presents the findings from the regression analyses assessing the moderating role of power distance in the relationship between boss phubbing, satisfaction with supervision (JDI), and job satisfaction. Additionally, the analysis examines the conditional indirect effects of boss phubbing on job satisfaction through satisfaction with supervision (JDI) at varying levels of power distance (refer Figure 2).

**Table 6:** Regression results for conditional indirect effect.

Predictor	*β*	SE	*t*	*p*	LCL	UCL
Outcome: job satisfaction						
Constant	3.5374	0.1627	21.743	0.000	3.217	3.857
Boss phubbing	−0.0302	0.0422	−0.717	0.473	−0.1131	0.052
Satisfaction with the supervision	0.2512	0427	5.8904	0.0000	0.1674	0.3351
Power distance	0.0445	0.0457	0.9739	0.3307	−0.0453	0.1342
Outcome: Satisfaction with the supervision						
Constant	3.628	0.059	61.115	0.000	3.511	3.744
Boss phubbing	0.386	0.045	−8.542	0.000	−0.475	−0.297
Power distance	−0.151	0.068	−2.202	0.028	−0.286	−0.016
Boss phubbing * Power distance	−0.047	0.050	−0.935	0.350	−0.146	0.052

Power distance	Boot indirect effect	Boot SE	LCL	UCL		
Conditional indirect effect at NTL = M ± 1SD						
−1SD (−0.874)	−0.0868	0.0223	−0.1365	−0.0484		
M (0.000)	−0.0972	0.0218	−0.1442	−0.0581		
+ 1SD (0.849)	−0.1073	0.0259	−0.1617	−0.0600		

*N* = 244. Unstandardized regression results are reported. Bootstrap sample size = 5.000. arange of values represent an abbreviated version of the output provided by macro. *p < 0.05.

The results indicate that boss phubbing is significantly and negatively associated with satisfaction with supervision (JDI) (*β* = −0.386, SE = 0.045, *t* = −8.542, *p* < 0.001), supporting H1. However, the direct relationship between boss phubbing and job satisfaction is not statistically significant (*β* = −0.0302, SE = 0.0422, *t* = −0.717, *p* = 0.473), providing no support for H2. In contrast, satisfaction with supervision (JDI) is significantly and positively associated with job satisfaction (*β* = 0.2512, SE = 0.0427, *t* = 5.8904, *p* < 0.001), confirming H3.

**Figure 3:**
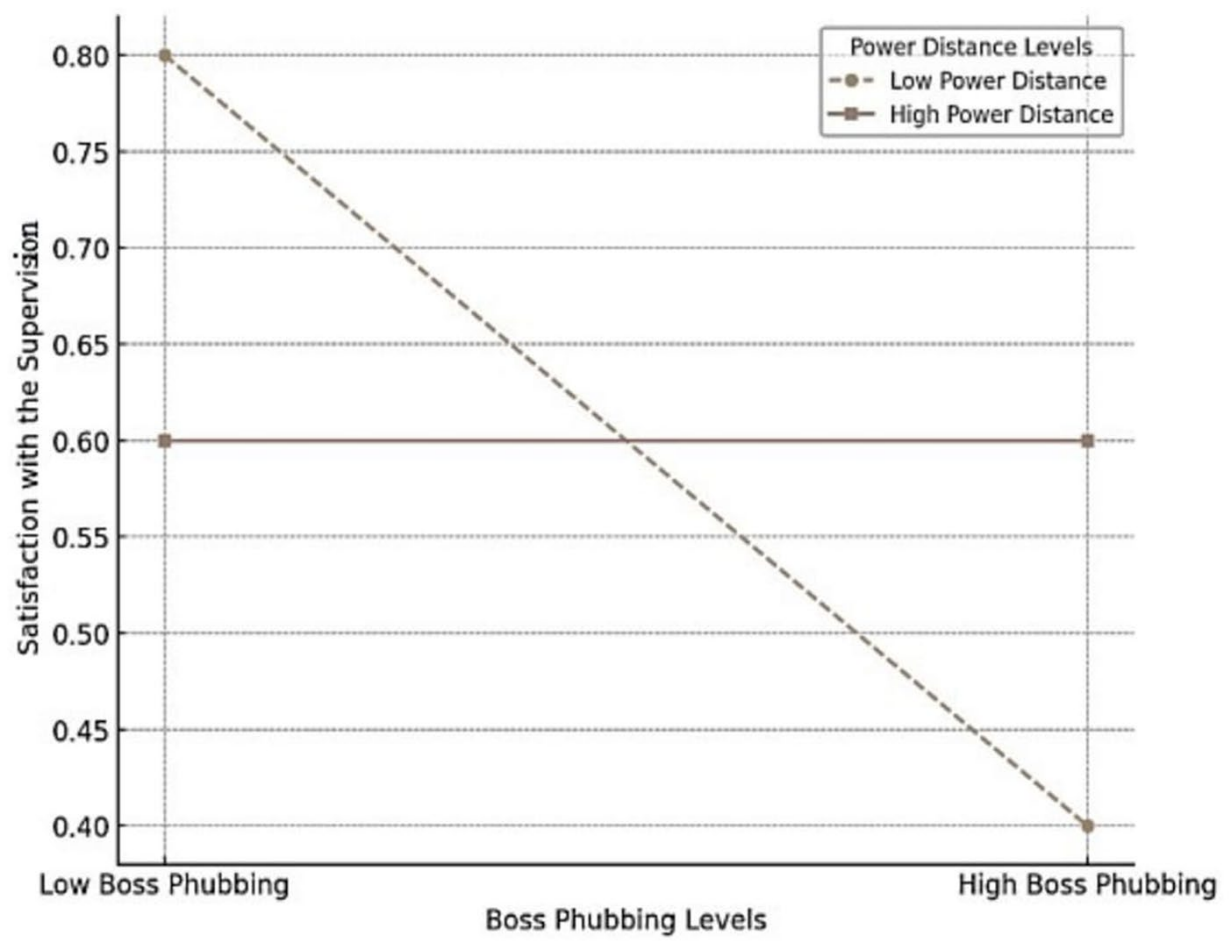
Moderating role of power distance on the relationship between boss phubbing and satisfaction with supervision (JDI).

The interaction term between boss phubbing and power distance was not statistically significant (*β* = −0.047, SE = 0.050, *t* = −0.935, *p* = 0.350), indicating that power distance does not significantly moderate the relationship between boss phubbing and satisfaction with supervision (JDI). As a result, H5 is not supported. However, a descriptive examination of the data (refer to Figure 3) suggests that the negative relationship between boss phubbing and satisfaction with supervision (JDI) may appear weaker for individuals in high power distance contexts compared to those in low power distance contexts.

The conditional indirect effects of boss phubbing on job satisfaction through satisfaction with supervision (JDI) were analyzed using the PROCESS Macro Model 7 with bootstrap confidence intervals. The analysis was conducted at three levels of power distance: one standard deviation below the mean (low power distance), the mean, and one standard deviation above the mean (high power distance). At low power distance (−1 SD), the indirect effect was significant [*β* = −0.0868, SE = 0.0223, 95% CI (−0.1365, −0.0484)]. At the mean level of power distance, the indirect effect remained significant [*β* = −0.0972, SE = 0.0218, 95% CI (−0.1442, −0.0581)]. At high power distance (+1 SD), the indirect effect was also significant [*β* = −0.1073, SE = 0.0259, 95% CI (−0.1617, −0.0600)].

**Figure 4:**
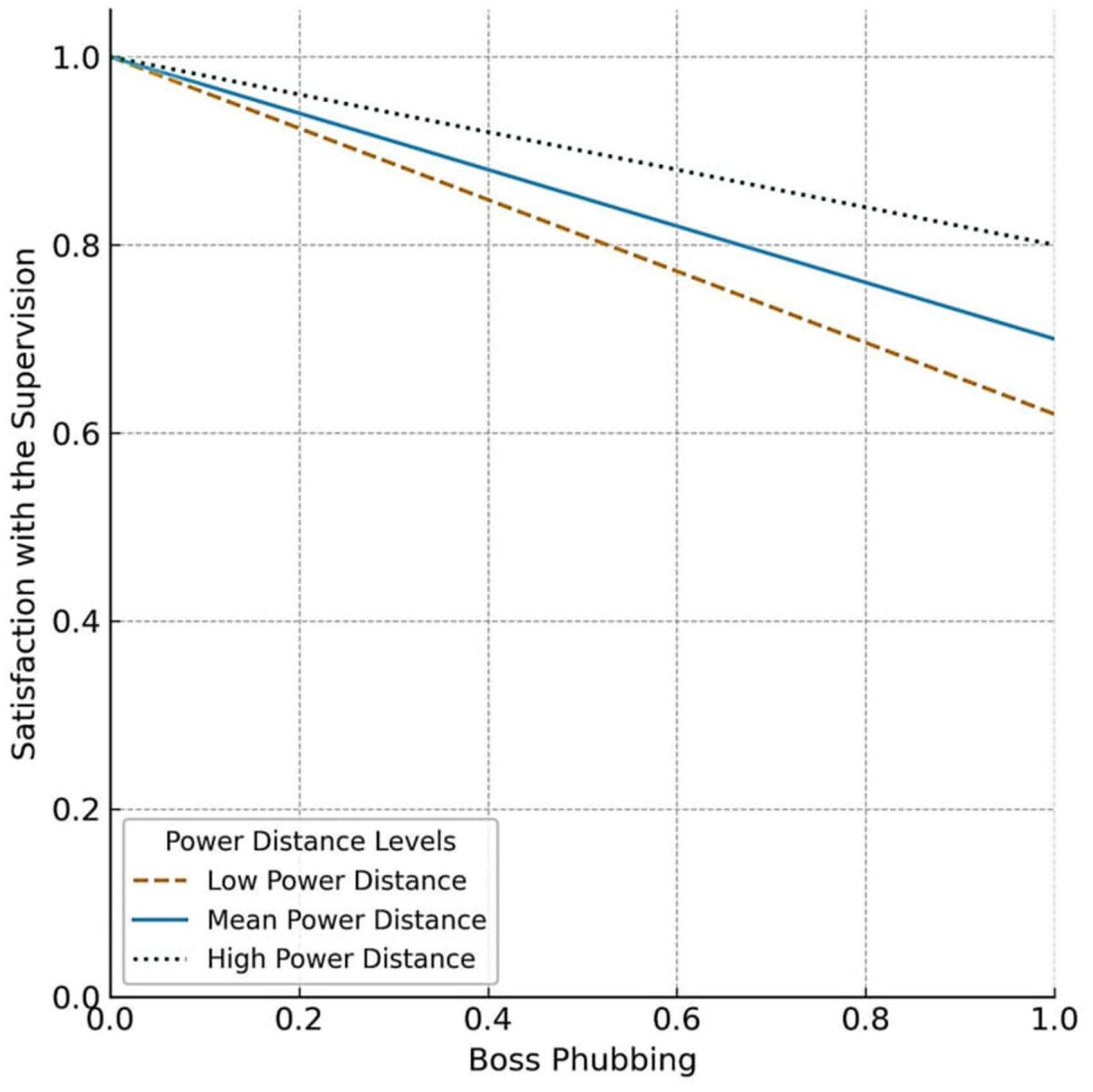
Satisfaction with supervision (JDI) predicted by boss-phubbing moderated by power distance.

The presence of significant conditional indirect effects despite a non-significant interaction effect is statistically plausible and reflects the dynamics of the model. Specifically, the interaction effect evaluates whether the direct relationship between boss phubbing and satisfaction with supervision (JDI) varies significantly across levels of power distance. In contrast, conditional indirect effects assess how the mediated relationship between boss phubbing and job satisfaction changes at specific levels of power distance (refer to Figure 4). A non-significant interaction effect indicates that moderation is not globally impactful, but conditional indirect effects can still be meaningful at specific levels of the moderator.

The PROCESS Macro uses bootstrap confidence intervals to evaluate conditional indirect effects. These intervals may reveal significant indirect effects at particular levels of power distance, even if the overall interaction effect lacks significance. While the interaction effect is not significant, the results suggest that power distance influences the strength of the mediated relationship (through satisfaction with supervision (JDI)) between boss phubbing and job satisfaction. This indicates that power distance may still play a meaningful, albeit nuanced, role in shaping the indirect effects.

Overall, the findings indicate that satisfaction with supervision (JDI) mediates the relationship between boss phubbing and job satisfaction, and that this mediated effect is significant across all levels of power distance. Although the interaction term was not statistically significant, the conditional indirect effects were consistently significant, providing partial support for H6. The pattern of conditional indirect effects is aligned with our theorizing, in that the indirect effect of BP on job satisfaction through satisfaction with supervision (JDI) is somewhat stronger at lower levels of power distance. However, the differences in effect size across levels of power distance are modest, and the moderated mediation should therefore be interpreted with caution.

## 5 Discussion

This study investigates the effects of BP—a supervisor’s behavior of ignoring or showing disinterest in employees by using a smartphone or other electronic devices—on employees’ satisfaction with their supervisor and job satisfaction, within the framework of power distance. The findings provide empirical evidence that BP diminishes both satisfaction with supervision (JDI) and job satisfaction, revealing the mechanism through which this occurs. Specifically, BP indirectly reduces job satisfaction via its negative impact on satisfaction with supervision (JDI).

Contrary to our initial expectation, BP is not directly associated with job satisfaction. This absence of a direct effect does not mean that BP is inconsequential; employees evaluate their work context holistically, and interactions with supervisors represent only one component of that broader appraisal. Our findings instead suggest that satisfaction with one’s supervisor serves as a key psychological mechanism through which BP ultimately shapes job satisfaction. This mechanism is consistent with evidence that the attitudinal consequences of boss phubbing operate via relational–affective pathways—such as work alienation—rather than through a direct effect on global job attitudes (Yao and Nie, 2023). It is also congruent with relational perspectives derived from Social Exchange Theory and Leader–Member Exchange, which emphasize that leaders influence job attitudes primarily through the quality of ongoing exchange relationships and the extent to which followers perceive themselves as valued and respected partners (Blau, 1964; Cropanzano and Mitchell, 2005; Graen and Uhl-Bien, 1995). In short, BP undermines employees’ perceptions of the supervisor as an attentive, fair, and supportive exchange partner, and this deterioration in satisfaction with supervision (JDI) subsequently translates into lower job satisfaction, reinforcing prior research linking satisfaction with supervision (JDI) to positive job attitudes, including organizational commitment (DeConinck and Stilwell, 2004). While this study did not directly assess perceived respect or the frustration of social needs, the pattern of results aligns with the interpretation that boss phubbing triggers an expectancy violation, weakening employees’ perceptions of interactional fairness and belonging, which in turn contributes to reduced satisfaction with supervision (JDI) and overall job satisfaction.

Moreover, the study does not find empirical support for the moderating role of power distance in the direct relationship between BP and satisfaction with supervision (JDI). From the perspective of EVT, this pattern suggests that the violation valence of boss phubbing is strongly negative across different levels of power distance. When a supervisor diverts attention from an employee to a smartphone during an interaction, this act typically violates basic expectations of attentiveness, respect, and interpersonal consideration. Regardless of employees’ endorsement of hierarchical relations, such behavior is likely interpreted as a devaluation of the focal interaction. At the same time, employees in high power distance contexts may attribute high communicator reward valence to their supervisors, viewing them as powerful gatekeepers of valued outcomes and career opportunities. As a result, they may suppress overt negative reactions and refrain from explicitly downgrading their evaluations of the supervisor, even when they experience BP as a negative event. These dynamics help explain why BP consistently undermines satisfaction with supervision (JDI), yet the strength of this direct effect does not systematically vary with power distance.

A contextual explanation is also consistent with the demographic profile of the sample, which predominantly comprised young and highly educated employees. These employees are embedded in workplaces shaped by globalized organizational practices and pervasive digital communication. They are therefore likely to frame BP primarily as unprofessional and inattentive managerial behavior, rather than as a culturally sanctioned leadership style. In line with emerging evidence that intercultural differences in basic expectations of respectful treatment are diminishing among Millennials and Gen Z employees, they may evaluate BP independently of traditional cultural prescriptions regarding deference to authority figures.

However, the findings suggest that power distance modestly qualifies the indirect relationship between BP and job satisfaction. The negative indirect effect of BP on job satisfaction through satisfaction with supervision (JDI) appears somewhat stronger in low power distance contexts and weaker in high power distance contexts. This pattern indicates that power distance operates more as a boundary condition for how dissatisfaction with the supervisor spills over into global job attitudes than for how BP is initially evaluated. Employees low in power distance hold stronger expectations for symmetrical, participatory relationships with their supervisors and expect their perspectives to be taken seriously. When these expectations are violated by BP, the negative violation valence is not offset by communicator reward valence, and diminished satisfaction with supervision (JDI) more readily translates into lower job satisfaction. By contrast, employees high in power distance are more accustomed to hierarchical distance and more likely to construe BP as part of the leader’s prerogative. They may cognitively rationalize or emotionally regulate their reactions, such that reduced satisfaction with supervision (JDI) has a weaker downstream impact on overall job satisfaction, even though BP still constitutes a negative relational event.

### 5.1 Theoretical contributions

This study makes several contributions to the literature on EVT, boss phubbing, and job attitudes. First, it identifies a relational–attitudinal mechanism linking boss phubbing to job satisfaction. Whereas prior research has primarily focused on BP’s effects on outcomes such as performance, psychological capital (Zhen and Wen, 2022), work meaningfulness (Khan et al., 2022), and trust in managers (Roberts and David, 2017), our findings show that BP indirectly undermines job satisfaction via satisfaction with supervision (JDI). In line with EVT, boss phubbing is conceptualized as a violation of employees’ expectations for attentiveness and respect, and our results demonstrate that this violation is translated into lower job satisfaction primarily through employees’ affective evaluations of their supervisor. By highlighting a relational–affective pathway, the study extends EVT into the domain of digital workplace behaviors and clarifies how seemingly mundane smartphone use by supervisors can erode global job attitudes through supervisor-focused evaluations (Yao and Nie, 2023; DeConinck and Stilwell, 2004).

Second, by integrating EVT with Social Exchange Theory and Leader–Member Exchange, the study refines theoretical assumptions about the role of supervisor behavior in shaping job attitudes. Boss phubbing is conceptualized not only as an expectancy violation but also as a negative relational signal that communicates low relational value and weak relational investment by the supervisor. From a SET and LMX perspective, such signals imply that the leader is not a fully engaged, fair, or supportive exchange partner (Blau, 1964; Cropanzano and Mitchell, 2005; Graen and Uhl-Bien, 1995). Our findings that BP consistently diminishes satisfaction with supervision (JDI) are consistent with this logic and suggest that the quality of ongoing leader–member exchanges is a key conduit through which digital interruptions are translated into job attitudes. At the same time, the absence of a moderating effect of power distance on the direct link between BP and satisfaction with supervision (JDI) indicates that managerial attentiveness has become a broadly shared expectation in contemporary organizations, rather than a preference confined to low power distance contexts. This pattern challenges the assumption that followers in high power distance environments are generally tolerant of intrusive, inattentive, or self-focused leadership behaviors and aligns with emerging cross-cultural evidence that certain core leadership behaviors—such as engagement and responsiveness—are valued across cultures (Rockstuhl et al., 2012; House et al., 2004).

Third, the study advances the literature by showing that power distance can function as a boundary condition for the indirect, rather than the direct, effect of boss phubbing on job satisfaction. The moderated mediation results indicate that power distance shapes the extent to which reduced satisfaction with supervision (JDI) spills over into global job attitudes, with a somewhat stronger indirect effect at lower levels of power distance. Although the differences in effect size are modest, the pattern is consistent with our cultural contingency arguments and highlights the value of examining cultural values as moderators of attitudinal pathways, not only of direct leader–outcome links. For employees high in power distance, hierarchical distance and perceived leader prerogatives weaken the translation of dissatisfaction with the supervisor into global job evaluations, even though BP is still experienced as a negative relational event. In EVT and SET terms, the combination of negative violation valence and high communicator reward valence leads high power distance employees to regulate, compartmentalize, or cognitively reframe their reactions, thereby attenuating the spillover from supervisor-focused attitudes to job satisfaction. These insights underscore the importance of treating cultural values as boundary conditions for the relational and attitudinal processes through which digital workplace behaviors such as boss phubbing exert their influence (Erdogan et al., 2006; Farh et al., 2007).

### 5.2 Practical implications

The evidence indicates that sustaining attentiveness in face-to-face interactions is not a peripheral interpersonal nicety but a core leadership competency with direct implications for employee attitudes. Organizations should therefore institutionalize prevention and development around boss phubbing (BP) within routine human-resource systems rather than treating it as an ad-hoc etiquette issue. In selection, supervisors should be evaluated not only on technical credentials but also on their discipline in managing attention under interruption pressure; behaviorally anchored prompts that elicit concrete decisions in phone-alert scenarios help surface this capacity. In development, generic awareness sessions are unlikely to shift behavior. Managers benefit more from practice-based interventions that make the relational costs of inattentiveness salient—e.g., structured role-plays that require them to experience a conversation from the employee’s vantage point and to rehearse attention-management routines in both physical and digital contexts.

Performance systems should translate values into observable criteria. Multi-source feedback can incorporate indicators of focused engagement during one-to-one exchanges and provide psychologically safe avenues for employees to report instances of inattentiveness as learning inputs rather than disciplinary triggers. Policy also has a role: clear expectations for mobile-phone use in high-stakes interactions (performance discussions, coaching, and feedback meetings) support perceptions of fairness and normalize digital etiquette as part of culture. More broadly, the pattern of findings suggests that the consequences of BP are relational rather than merely evaluative. Interventions that strengthen leader–member relationship quality—coaching, communication training, and shared norms of presence—are therefore likely to outperform generic “device-restriction” rules, because they target the interpersonal mechanisms through which BP affects satisfaction with supervision (JDI).

### 5.3 Limitations and future research

Several limitations qualify the inferences that can be drawn from this study and point to promising directions for future work. First, the cross-sectional design constrains causal claims among BP, satisfaction with supervision (JDI), and job satisfaction. Longitudinal designs, experience-sampling studies, or field experiments would allow stronger inferences about temporal ordering and change dynamics, especially for downstream outcomes such as organizational commitment and performance. Second, because focal variables were self-reported, common method bias (CMB) was a potential concern. Harman’s single-factor test indicated that the first factor accounted for 23.42% of total variance—well below the 50% benchmark—suggesting CMB is unlikely to be severe; nonetheless, multi-source or time-lagged designs (e.g., supervisor and peer ratings) would further reinforce robustness.

Third, generalizability is bounded by context. The sample comprised primarily young, educated employees in Türkiye; cultural norms around hierarchy may shape how BP is appraised in workplace interactions. Replications across national and organizational cultures are needed to assess scope conditions. Fourth, the model did not include work engagement. In higher power-distance settings, BP may erode engagement rather than directly depressing satisfaction with supervision (JDI); employees who maintain positive surface evaluations of their supervisors may instead withdraw psychologically from work. Incorporating engagement and other proximal motivational states would clarify whether they constitute alternative or complementary pathways to job satisfaction, commitment, and performance.

Fifth, the underlying socio-relational mechanisms merit direct testing. Future research should examine whether perceived respect and relatedness-need fulfillment mediate the effects of BP on employee attitudes, thereby providing stronger process evidence. Sixth, measurement breadth remains limited. Scale refinement that differentiates visibility from active use during interaction, attends to timing and context, and improves sensitivity to event-level variance would expand methodological leverage. Finally, while supervisors may at times use smartphones for instrumental, work-related purposes, the present account—consistent with EVT—centers on employees’ perceptions of inattentiveness in face-to-face encounters. Future studies should explicitly measure perceived justification or instrumentality and test whether such appraisals attenuate reactions to BP. Beyond power distance, examining additional cultural dimensions (e.g., individualism–collectivism, uncertainty avoidance, and masculinity–femininity) within cross-level models would further illuminate when and for whom BP is most consequential.

## 6 Conclusion

This study contributes to organizational behavior research by examining the impact of boss phubbing on job satisfaction through satisfaction with supervision (JDI) and power distance. By extending EVT, it underscores the critical role of managerial attentiveness in shaping employee experiences and workplace outcomes. The findings highlight that BP indirectly undermines job satisfaction through its negative effect on satisfaction with supervision (JDI), and that cultural factors, particularly power distance, moderate this indirect relationship.

Future research should continue to investigate cultural variations in managerial behavior and the implications of digital distractions in the workplace, offering deeper insights into how employees across different cultural contexts respond to technology-driven disruptions in leadership interactions.

## Data Availability

The raw data supporting the conclusions of this article will be made available by the authors, without undue reservation.

## References

[ref1] Aagaard J. (2020). Digital akrasia: a qualitative study of phubbing. AI Soc. 35, 237–244. doi: 10.1007/s00146-019-00876-0

[ref2] Abeele M. M. V. Hendrickson A. T. Pollmann M. M. Ling R. (2019). Phubbing behavior in conversations and its relation to perceived conversation intimacy and distraction: an exploratory observation study. Comput. Hum. Behav. 100, 35–47. doi: 10.1016/j.chb.2019.06.004

[ref3] Adebayo S. O. Ogunsina S. O. (2011). Influence of supervisory behaviour and job stress on job satisfaction and turnover intention of police personnel in Ekiti state. J. Manag. Strateg. 2, 13–20. doi: 10.5430/jms.v2n3p13

[ref4] Alegre I. Mas-Machuca M. Berbegal-Mirabent J. (2016). Antecedents of employee job satisfaction: do they matter? J. Bus. Res. 69, 1390–1395. doi: 10.1016/j.jbusres.2015.10.113

[ref5] Al-Saggaf Y. MacCulloch R. (2019). Phubbing and social relationships: results from an Australian sample. J. Relat. Res. 10, 1–10. doi: 10.1017/jrr.2019.9, 41306117

[ref6] Anshari M. Alas Y. Hardaker G. Jaidin J. H. Smith M. Ahad A. D. (2016). Smartphone habit and behavior in Brunei: personalization, gender, and generation gap. Comput. Hum. Behav. 64, 719–727. doi: 10.1016/j.chb.2016.07.063

[ref7] Babalola S. S. (2016). The effect of leadership style, job satisfaction and employee-supervisor relationship on job performance and organizational commitment. J. Appl. Bus. Res. 32, 935–946. doi: 10.19030/jabr.v32i3.9667

[ref8] Bano S. Ashraf N. Shahzadi K. (2024). Impact of perceived boss phubbing on employee work alienation and employee presenteeism through the mediating role of organizational pride. Pak. Soc. Sci. Rev. 8, 99–111. doi: 10.35484/pssr.2024(8-II)09

[ref9] Becker T. E. Atinc G. Breaugh J. A. Carlson K. D. Edwards J. R. Spector P. E. (2016). Statistical control in correlational studies: 10 essential recommendations for organizational researchers. J. Organ. Behav. 37, 157–167. doi: 10.1002/job.2053

[ref10] Belias D. Koustelıos A. 2014 Organizational culture and job satisfaction: a review” Int. Rev. Manag. Mark. 4 132–149. Available online at: https://www.econjournals.com/index.php/irmm/article/view/746

[ref11] Bernerth J. B. Aguinis H. (2016). A critical review and best-practice recommendations for control variable usage. Pers. Psychol. 69, 229–283. doi: 10.1111/peps.12103

[ref12] Blau P. M. (1964). Exchange and power in social life. New York, NY: Wiley.

[ref13] Bracht E. M. Hernandez Bark A. S. She Z. Van Dick R. Junker N. M. (2024). The downside of phones at work: exploring negative relationships between leader phubbing and follower engagement/performance. Leadersh. Organ. Dev. J. 45, 82–93. doi: 10.1108/LODJ-03-2023-0129

[ref14] Brandt R. B. (1979). A theory of the good and the right. Oxford: Clarendon Press.

[ref15] Brayfield A. H. Rothe H. F. (1951). An index of job satisfaction. J. Appl. Psychol. 35, 307–311. doi: 10.1037/h0055617

[ref16] Brockner J. Ackerman G. Greenberg J. Gelfand M. J. Francesco A. M. Chen Z. X. . (2001). Culture and procedural justice: the influence of power distance on reactions to voice. J. Exp. Soc. Psychol. 37, 300–315. doi: 10.1006/jesp.2000.1451

[ref17] Burgoon J. K. (1993). Interpersonal expectations, expectancy violations, and emotional communication. J. Lang. Soc. Psychol. 12, 30–48. doi: 10.1177/0261927X931210

[ref18] Burgoon J. K. (2015). “Expectancy violations theory” in The international encyclopedia of interpersonal communication. eds. Berger C. R. Roloff M. E. Wilson S. R. Dillard J. P. Caughlin J. Solomon D. (Malden, MA: John Wiley and Sons).

[ref19] Burgoon J. K. Hale J. L. (1988). Nonverbal expectancy violations: model elaboration and application to immediacy behaviors. Commun. Monogr. 55, 58–79. doi: 10.1080/03637758809376158

[ref20] Burgoon J. K. Hubbard A. S. E. (2005). “Cross-cultural and intercultural applications of expectancy violations theory and interaction adaptation theory” in Theorizing about intercultural communication. ed. Gudykunst W. B. (Thousands Oaks, California: Sage Publications), 149–171.

[ref21] Burgoon J. K. Le Poire B. A. (1993). Effects of communication expectancies, actual communication, and expectancy disconfirmation on evaluations of communicators and their communication behavior. Hum. Commun. Res. 20, 67–96. doi: 10.1111/j.1468-2958.1993.tb00316.x

[ref22] Burgoon J. K. Newton D. A. Walther J. A. Baesler E. J. (1989). Nonverbal expectancy violations and conversational involvement. J. Nonverbal Behav. 13, 97–119. doi: 10.1111/j.1468-2958.1989.tb00210.x

[ref23] Çakmak E. Öztekin Ö. Karadağ E. (2015). “The effect of leadership on job satisfaction” in Leadership and organizational outcomes. ed. Karadağ E. (Switzerland: Springer).

[ref24] Career Builder 2016 New CareerBuilder survey reveals how much smartphones are sapping productivity at work. Available online at: https://www.prnewswire.com/news-releases/new-careerbuilder-survey-reveals-how-much-smartphones-are-sapping-productivity-at-work-300281718.html#:~:text=More%20than%208%20in%2010,times%20a%20day%20while%20working (Accessed September 10, 2024).

[ref25] Castañeda M. Nahavandi A. (1991). Link of manager behavior to supervisor performance rating and subordinate satisfaction. Group Organ. Stud. 16, 357–366. doi: 10.1177/105960119101600402

[ref26] Chotpitayasunondh V. Douglas K. M. (2016). How “phubbing” becomes the norm: the antecedents and consequences of snubbing via smartphone. Comput. Hum. Behav. 63, 9–18. doi: 10.1016/j.chb.2016.05.018

[ref27] Chotpitayasunondh V. Douglas K. M. (2018). The effects of “phubbing” on social interaction. J. Appl. Soc. Psychol. 48, 304–316. doi: 10.1111/jasp.12506

[ref28] Çikrikci Ö. Griffiths M. D. Erzen E. (2019). Testing the mediating role of phubbing in the relationship between the big five personality traits and satisfaction with life. Int. J. Ment. Health Addict. 20, 44–56. doi: 10.1007/s11469-019-00115-z

[ref29] Cropanzano R. Mitchell M. S. (2005). Social exchange theory: an interdisciplinary review. J. Manag. 31, 874–900. doi: 10.1177/0149206305279602

[ref30] Czech K. Forward G. L. (2013). Communication, leadership, and job satisfaction: perspectives on supervisor-subordinate relationships. Stud. Media Commun. 1, 11–24. doi: 10.11114/smc.v1i2.122, 39100657

[ref31] DeConinck J. B. Stilwell C. D. (2004). Incorporating organizational justice, role states, pay satisfaction and satisfaction with supervision (JDI) in a model of turnover intentions. J. Bus. Res. 57, 225–231. doi: 10.1016/S0148-2963(02)00289-8

[ref32] Derks D. Bakker A. B. (2014). Smartphone use, work–home interference, and burnout: a diary study on the role of recovery. Appl. Psychol. 63, 411–440. doi: 10.1111/j.1464-0597.2012.00530.x

[ref33] Dirks K. T. Ferrin D. L. (2001). The role of trust in organizational settings. Organ. Sci. 12, 450–467. doi: 10.1287/orsc.12.4.450.10640, 19642375

[ref34] Edwards J. R. Lambert L. S. (2007). Methods for integrating moderation and mediation: a general analytical framework using moderated path analysis. Psychol. Methods 12, 1–22. doi: 10.1037/1082-989X.12.1.1, 17402809

[ref35] Egerová D. Komárková L. Kutlák J. (2021). Generation Y and generation Z employment expectations: a generational cohort comparative study from two countries. E + M Ekon. Manag. 24, 93–109. doi: 10.15240/tul/001/2021-03-006

[ref36] Erdogan B. Liden R. C. Kraimer M. L. (2006). Justice and leader-member exchange: the moderating role of organizational culture. Acad. Manag. J. 49, 395–406. doi: 10.5465/amj.2006.20786086

[ref37] Erzen E. Odaci H. Yeniçeri İ. (2019). Phubbing: which personality traits are prone to phubbing? Soc. Sci. Comput. Rev. 39, 56–69. doi: 10.1177/0894439319847415, 41321435

[ref38] Farh J. L. Hackett R. D. Liang J. (2007). Individual-level cultural values as moderators of perceived organizational support–employee outcome relationships in China: comparing the effects of power distance and traditionality. Acad. Manag. J. 50, 715–729. doi: 10.5465/amj.2007.25530866

[ref39] Fornell C. Larcker D. F. (1981). Evaluating structural equation models with unobservable variables and measurement error. J. Mark. Res. 18, 39–50. doi: 10.1177/002224378101800

[ref40] García-Cabrera A. M. Suárez-Ortega S. M. Gutiérrez-Pérez F. J. Miranda-Martel M. J. (2023). The influence of supervisor supportive behaviors on subordinate job satisfaction: the moderating effect of gender similarity. Front. Psychol. 14:1233212. doi: 10.3389/fpsyg.2023.1233212, 38222844 PMC10785647

[ref41] Graen G. B. Uhl-Bien M. (1995). Relationship-based approach to leadership: development of leader–member exchange (LMX) theory over 25 years: applying a multi-level multi-domain perspective. Leadersh. Q. 6, 219–247. doi: 10.1016/1048-9843(95)90036-5

[ref42] Griffin M. A. Patterson M. G. West M. A. (2001). Job satisfaction and teamwork: the role of supervisor support. J. Organ. Behav. 22, 537–550. doi: 10.1002/job.101

[ref43] Hair J. F. Black W. C. Babin B. J. Anderson R. E. (2010). Multivariate data analysis. New York: Prentice Hall.

[ref44] Han G. Jekel M. (2011). The mediating role of job satisfaction between leader-member exchange and turnover intentions. J. Nurs. Manag. 19, 41–49. doi: 10.1111/j.1365-2834.2010.01184.x, 21223404

[ref45] Hasan S. A. Naseem A. Mahmood M. Sajjad Z. Mirza M. Z. (2024). Impact of supervisor phubbing on workplace incivility and workplace presenteeism: mediation and moderation effect of self-esteem and power distance. J. Manag. Dev. 43, 68–86. doi: 10.1108/JMD-08-2023-0230

[ref46] Hayes A. F. (2012). PROCESS: a versatile computational tool for observed variable mediation, moderation, and conditional process modeling. [White paper]. Available at http://www.afhayes.com/public/process (Accessed April 03, 2025).

[ref47] Hayes A. F. (2013). Introduction to mediation, moderation, and conditional process analysis: A regression-based approach. New York: Guilford Press.

[ref48] Hofstede G. (1980). Culture and organizations. Int. Stud. Manag. Organ. 10, 15–41. doi: 10.1080/00208825.1980.11656300

[ref49] Høgh A. Clausen T. Bickmann L. Hansen Å. M. Conway P. M. Baernholdt M. (2021). “Consequences of workplace bullying for individuals, organizations and society” in Pathways of job-related negative behaviour. ed. Cruz P. D. (Singapore: Springer Nature), 177–200.

[ref50] House R. J. Hanges P. J. Javidan M. Dorfman P. W. Gupta V. (2004). Culture, leadership, and organizations: the GLOBE study of 62 societies. Thousand Oaks, CA: Sage Publications.

[ref51] Jehanzeb K. Mohanty J. (2020). The mediating role of organizational commitment between organizational justice and organizational citizenship behavior: power distance as moderator. Pers. Rev. 49, 445–468. doi: 10.1108/PR-09-2018-0327

[ref52] Karadağ E. Tosuntaş Ş. B. Erzen E. Duru P. Bostan N. Şahin B. M. (2015). Determinants of phubbing, which is the sum of many virtual addictions: a structural equation model. J. Behav. Addict. 4, 60–74. doi: 10.1556/2006.4.2015.005, 26014669 PMC4500886

[ref53] Khan M. N. Shahzad K. Ahmad I. Bartels J. (2023). Boss, look at me: how and when supervisor’s phubbing behavior affects employees’ supervisor identification. Curr. Psychol. 42, 31064–31078. doi: 10.1007/s12144-022-04120-9

[ref54] Khan M. N. Shahzad K. Bartels J. (2022). Examining boss phubbing and employee outcomes through the lens of affective events theory. Aslib J. Inf. Manag. 74, 877–900. doi: 10.1108/AJIM-07-2021-0198

[ref55] Lee Y. T. Antonakis J. (2014). When preference is not satisfied but the individual is: how power distance moderates person–job fit. J. Manag. 40, 641–675. doi: 10.1177/01492063114360

[ref56] Liu K. Chen W. Wang H. Geng J. Lei L. (2021). Parental phubbing linking to adolescent life satisfaction: the mediating role of relationship satisfaction and the moderating role of attachment styles. Child Care Health Dev. 47, 281–289. doi: 10.1111/cch.12839, 33314201

[ref57] Locke E. A. (1969). What is job satisfaction? Organ. Behav. Hum. Perform. 4, 309–336. doi: 10.1016/0030-5073(69)90013-0

[ref58] Mihalcea A. (2014). Leadership, personality, job satisfaction and job performance. Procedia Soc. Behav. Sci. 127, 443–447. doi: 10.1016/j.sbspro.2014.03.287

[ref59] Miles E. W. Patrick S. L. King W. C. Jr. (1996). Job level as a systemic variable in predicting the relationship between supervisory communication and job satisfaction. J. Occup. Organ. Psychol. 69, 277–292. doi: 10.1111/j.2044-8325.1996.tb00615.x

[ref60] Mishra P. K. (2013). Job satisfaction. IOSR J. Humanit. Soc. Sci. 14, 45–54. doi: 10.9790/1959-1454554

[ref61] MOBİSAD (2023). Türkiye’nin ve dünyanın dijital karnesi. Mobil İletişim Sektörü Raporu. Available online at: https://mobisad.org/wp-content/uploads/rapor-2023.pdf (Accessed January 15, 2025).

[ref62] Özdemir S. (2020). Yönetici sosyotelizmi (phubbing): Bir ölçek uyarlama çalişmasi. Dicle Üniversitesi İktisadi ve İdari Bilimler Fakültesi Dergisi 10, 134–145.

[ref63] Platis C. Reklitis P. Zimeras S. (2015). Relation between job satisfaction and job performance in healthcare services. Procedia Soc. Behav. Sci. 175, 480–487. doi: 10.1016/j.sbspro.2015.01.1226

[ref64] Podsakoff P. M. Organ D. W. (1986). Self-reports in organizational research: problems and prospects. J. Manag. 12, 531–544. doi: 10.1177/014920638601200

[ref65] Pohl S. Galletta M. (2017). The role of supervisor emotional support on individual job satisfaction: a multilevel analysis. Appl. Nurs. Res. 33, 61–66. doi: 10.1016/j.apnr.2016.10.004, 28096025

[ref66] Preacher K. J. Rucker D. D. Hayes A. F. (2007). Addressing moderated mediation hypotheses: theory, methods, and prescriptions. Multivar. Behav. Res. 42, 185–227. doi: 10.1080/00273170701341316, 26821081

[ref67] Riaz A. Haider M. H. (2010). Role of transformational and transactional leadership on job satisfaction and career satisfaction. Bus. Econ. Horiz. 1, 29–38. doi: 10.15208/beh.2010.05, 40696330

[ref68] Ríos Ariza J. M. Matas-Terrón A. del Rumiche Chávarry R. P. Chunga Chinguel G. R. (2021). Scale for measuring phubbing in Peruvian university students: adaptation, validation and results of its application. J. New Approaches Educ. Res. 10, 175–189. doi: 10.7821/naer.2021.7.606

[ref69] Roberts J. A. David M. E. (2016). My life has become a major distraction from my cell phone: partner phubbing and relationship satisfaction among romantic partners. Comput. Human Behav. 54, 134–141. doi: 10.1016/j.chb.2015.07.058

[ref70] Roberts J. A. David M. E. (2017). Put down your phone and listen to me: how boss phubbing undermines the psychological conditions necessary for employee engagement. Comput. Human Behav. 75, 206–217. doi: 10.1016/j.chb.2017.05.021

[ref71] Roberts J. A. David M. E. (2020). Boss phubbing, trust, job satisfaction and employee performance. Pers. Individ. Differ. 155:109702. doi: 10.1016/j.paid.2019.109702

[ref72] Rockstuhl T. Dulebohn J. H. Ang S. Shore L. M. (2012). Leader–member exchange (LMX) and culture: a meta-analysis of correlates of LMX across 23 countries. J. Appl. Psychol. 97, 1097–1130. doi: 10.1037/a0029978, 22985117

[ref73] Rowold J. Borgmann L. Bormann K. (2014). Which leadership constructs are important for predicting job satisfaction, affective commitment, and perceived job performance in profit versus nonprofit organizations? Nonprofit Manag. Leadersh. 25, 147–164. doi: 10.1002/nml.21116

[ref74] Roy I. Islam R. Arefin M. S. Rahman S. (2025). How perceived supervisor and organizational support shape job satisfaction: the intervening role of work-life balance and organizational identification. Open J. Bus. Manag. 13, 2782–2809. doi: 10.4236/ojbm.2025.134148

[ref75] Saputra D. Arief M. Gharnaditya D. Vhany D. 2018 Mediating effect of job satisfaction on relation between power distance and collectivism toward employee performance in Indonesia Pertanika J. Soc. Sci. Humanit. 26 75–86. Available online at: http://www.pertanika.upm.edu.my/pjtas/browse/regular-issue?article=JSSH-T0665-2018

[ref76] Schneider F. M. Hitzfeld S. (2021). I ought to put down that phone but I phub nevertheless: examining the predictors of phubbing behavior. Soc. Sci. Comput. Rev. 39, 1075–1088. doi: 10.1177/089443931988236

[ref77] Scholl M. D. (2003). Confluence: leadership, collegiality, good fortune. Harmony. 16, 96–104.

[ref78] Schwepker J. (2001). Ethical climate's relationship to job satisfaction, organizational commitment, and turnover intention in the salesforce. J. Bus. Res. 54, 39–52. doi: 10.1016/S0148-2963(00)00125-9

[ref79] Shahbaz K. Rasul F. Khan A. Saba A. Saeed M. Nida A. . (2020). Phubbing positively predicts psychological distress and poor quality of life in community adults. Int. J. Manag. 11, 2229–2240.

[ref80] Shrout P. E. Bolger N. (2002). Mediation in experimental and nonexperimental studies: new procedures and recommendations. Psychol. Methods 7, 422–445. doi: 10.1037/1082-989X.7.4.422, 12530702

[ref81] Singh S. K. (2024). Impact of supervisor’s interactional justice and subordinate outcomes. Br. J. Manag. 35, 1296–1312. doi: 10.1111/1467-8551.12758

[ref82] Smith P. Kendall L. F. Hulin C. (1969). The measurement of satisfaction in work and retirement. Chicago, IL,USA: Rand MC Nally.

[ref83] Spector P. E. Brannick M. T. (2011). Methodological urban legends: the misuse of statistical control variables. Organ. Res. Methods 14, 287–305. doi: 10.1177/1094428110369842

[ref84] Steele G. Plenty D. (2014). Supervisor–subordinate communication competence and job and communication satisfaction. Int. J. Bus. Commun. 52, 294–318. doi: 10.1177/2329488414525450

[ref85] Stringer L. (2006). The link between the quality of the supervisor–employee relationship and the level of the employee's job satisfaction. Public Organ. Rev. 6, 125–142. doi: 10.1007/s11115-006-0005-0

[ref86] Sun J. Samp J. A. (2022). ‘Phubbing is happening to you’: examining predictors and effects of phubbing behaviour in friendships. Behav. Inf. Technol. 41, 2691–2704. doi: 10.1080/0144929X.2021.1943711

[ref87] Tepret N. Y. Tuna K. (2015). Effect of management factor on employee job satisfaction: an application in telecommunication sector. Procedia. Soc. Behav. Sci. 195, 673–679. doi: 10.1016/j.sbspro.2015.06.264

[ref88] Teven J. J. (2007). Effects of supervisor social influence, nonverbal immediacy, and biological sex on subordinates' perceptions of job satisfaction, liking, and supervisor credibility. Commun. Q. 55, 155–177. doi: 10.1080/01463370601036036

[ref1101] Turkle S. (2011). Alone Together: Why We Expect More from Technology and Less from Each Other. New York: Basic Books.

[ref89] Ugur N. G. Koc T. (2015). Time for digital detox: misuse of mobile technology and phubbing. Procedia. Soc. Behav. Sci. 195, 1022–1031. doi: 10.1016/j.sbspro.2015.06.491

[ref90] Van der Wal M. A. Schönrock-Adema J. Scheele F. Schripsema N. R. Jaarsma A. D. C. Cohen-Schotanus J. (2016). Supervisor leadership in relation to resident job satisfaction. BMC Med. Educ. 16, 194–197. doi: 10.1186/s12909-016-0688-z, 27480528 PMC4968444

[ref91] Wang S. Fränti P. (2022). How power distance affect motivation in cross-cultural environment: findings from Chinese companies in Europe. STEM Educ. 2, 96–120. doi: 10.3934/steme.2022008

[ref92] Wang X. Xie X. Wang Y. Wang P. Lei L. (2017). Partner phubbing and depression among married Chinese adults: the roles of relationship satisfaction and relationship length. Pers. Individ. Differ. 110, 12–17. doi: 10.1016/j.paid.2017.01.014

[ref93] Xie X. Xie J. (2020). Parental phubbing accelerates depression in late childhood and adolescence: a two-path model. J. Adolesc. 78, 43–52. doi: 10.1016/j.adolescence.2019.12.004, 31821961

[ref94] Yao S. Nie T. (2023). Boss, can’t you hear me? The impact mechanism of supervisor phone snubbing (phubbing) on employee psychological withdrawal behavior. Healthcare 11:3167. doi: 10.3390/healthcare11243167, 38132057 PMC10742795

[ref95] Yasin R. M. (2021). Phubbing in the workplace: exploring the role of rejection sensitivity in the relationship between supervisor phubbing and employee outcomes. Bexley: Capital University.

[ref96] Yasin R. M. Bashir S. Abeele M. V. Bartels J. (2023). Supervisor phubbing phenomenon in organizations: determinants and impacts. Int. J. Bus. Commun. 60, 150–172. doi: 10.1177/232948842090712

[ref97] Yousaf S. Rasheed M. I. Kaur P. Islam N. Dhir A. (2022). The dark side of phubbing in the workplace: investigating the role of intrinsic motivation and the use of enterprise social media (ESM) in a cross-cultural setting. J. Bus. Res. 143, 81–93. doi: 10.1016/j.jbusres.2022.01.043

[ref98] Yuda I. H. Suyono J. (2024). Systematic literature review of boss phubbing from 2013–2023. J. Syntax Transform. 5, 406–415. doi: 10.46799/jst.v5i2.913

[ref99] Yurtseven C. N. Duman F. K. (2021). Evaluation of boss phubbing in sports businesses. Pak. J. Med. Health Sci. 15, 839–844.

[ref100] Zhen Y. Wen C. Y.. 2022. The impact of boss phubbing on employees’ job performance: a mediation model with moderation, in 2022 7th international conference on robotics and automation engineering (ICRAE) (pp. 384–387), IEEE.

